# Start Me Up: How Can Surrounding Gangliosides Affect Sodium-Potassium ATPase Activity and Steer towards Pathological Ion Imbalance in Neurons?

**DOI:** 10.3390/biomedicines10071518

**Published:** 2022-06-27

**Authors:** Borna Puljko, Mario Stojanović, Katarina Ilic, Svjetlana Kalanj-Bognar, Kristina Mlinac-Jerkovic

**Affiliations:** 1 Croatian Institute for Brain Research, School of Medicine, University of Zagreb, 10 000 Zagreb, Croatia; borna.puljko@mef.hr (B.P.); mario.stojanovic@mef.hr (M.S.); katarina.ilic@kcl.ac.uk (K.I.); svjetlana.kalanj.bognar@mef.hr (S.K.-B.); 2 BRAIN Centre, Department of Neuroimaging, Institute of Psychiatry, Psychology and Neuroscience (IOPPN), King’s College London, London SE5 9NU, UK

**Keywords:** glycosphingolipids, lipid rafts, membrane microdomains, neuronal ion homeostasis, GM1, Na^+^/K^+^-ATPase, sodium-potassium pump, epilepsy

## Abstract

Gangliosides, amphiphilic glycosphingolipids, tend to associate laterally with other membrane constituents and undergo extensive interactions with membrane proteins in *cis* or *trans* configurations. Studies of human diseases resulting from mutations in the ganglioside biosynthesis pathway and research on transgenic mice with the same mutations implicate gangliosides in the pathogenesis of epilepsy. Gangliosides are reported to affect the activity of the Na^+^/K^+^-ATPase, the ubiquitously expressed plasma membrane pump responsible for the stabilization of the resting membrane potential by hyperpolarization, firing up the action potential and ion homeostasis. Impaired Na^+^/K^+^-ATPase activity has also been hypothesized to cause seizures by several mechanisms. In this review we present different epileptic phenotypes that are caused by impaired activity of Na^+^/K^+^-ATPase or changed membrane ganglioside composition. We further discuss how gangliosides may influence Na^+^/K^+^-ATPase activity by acting as lipid sorting machinery providing the optimal stage for Na^+^/K^+^-ATPase function. By establishing a distinct lipid environment, together with other membrane lipids, gangliosides possibly modulate Na^+^/K^+^-ATPase activity and aid in “starting up” and “turning off” this vital pump. Therefore, structural changes of neuronal membranes caused by altered ganglioside composition can be a contributing factor leading to aberrant Na^+^/K^+^-ATPase activity and ion imbalance priming neurons for pathological firing.

## 1. Membrane Dynamics and Lipid-Protein Interplay

The plasma membrane is a highly complex and dynamic structure, organized as an asymmetric lipid bilayer containing various (macro)molecules including phospholipids, cholesterol, glycolipids, sphingolipids, and proteins. Membrane dynamics depend on membrane composition and the formation of liquid-ordered and disordered phases which accommodate specific proteins [1,2]. Proteins and lipids, as major constituents of biological membranes, undergo a number of interactions which underlie crucial phenomena and cellular functions such as preservation of membrane properties, signal transduction and molecular exchange of the cell with its environment [3]. Proper functions of proteins, the working part of the membrane assemblage, are intertwined with and influenced by the specific lipid environment and topology of the membrane microdomains. Both protein structures and functions are known to be regulated by specific interactions with their neighboring lipids [4,5,6], yet exact mechanisms are still poorly understood; hence, more detailed studies are required [7]. Recently, with the advances in structural biology, membrane proteins got their spotlight under the cryo-electron microscope and more innately bound lipids arose in structural models as crucial players in the regulation of membrane protein distribution and functions [8]. This opened the door for a detailed investigation in the field of protein-lipid interplay and allowed computational approaches to this matter. Molecular dynamics (MD) simulations have been used to study the protein-lipid interplay [9,10], by allowing membrane protein structures to be computationally assembled into lipid bilayers and their interactions characterized [11]. This approach can also take into consideration the glycan moieties of both protein and lipid constituents of the membrane. Amongst proteins readily investigated by these approaches are ion channels [12,13,14] and outer membrane proteins [15,16]. MD simulations have characterized experimentally observed interactions between cholesterol and G-protein coupled receptors (GPCRs) [10]. In addition, MD simulations have also been used to identify binding sites for phosphatidylinositol 4,5-bisphosphate (PIP_2_) in ion channels, transporters and receptor proteins [17]. MD became the method of choice for unraveling nanoscale phenomena since it yields great accuracy in understanding the properties of lipid membranes and lipid−protein interactions that can describe biological membranes in a relatively realistic manner [18]. Nevertheless, although computational models have provided important insight into the organization of plasma membrane and protein-lipid interactions, and interesting and relevant work has been performed on artificial membranes [19,20,21], they do not necessarily give an entirely accurate insight into protein-lipid interactions as they happen in membranes of living organisms.

Studying the physiological effect of lipids on membrane proteins and enzymes in actual “real” membranes, albeit challenging, is indispensable for thorough understanding of all membrane-related phenomena. The innovations in imaging techniques allow for detailed and accurate visualization of membrane dynamics in membrane systems and live cells [22,23,24]. For example, such visualisation showed that ganglioside GT1b together with synaptotagmin 1/2 serves as a receptor component for botulinum neurotoxin type B (BoNT/B) [25].

## 2. Gangliosides

### 2.1. Short Overwiev of Gangliosides

Glycolipids are amphiphilic molecules composed of hydrophobic alkyl tails, which anchor them to the phospholipid bilayer, and a hydrophilic oligosaccharide headgroup. Numerous species of glycolipids exist, differing in oligosaccharide headgroups, lipid backbones, and alkyl tails [26]. Gangliosides are glycosphingolipids consisting of a ceramide tail attached by a glycosidic linkage to a glycan headgroup with one or more sialic acid residues [27]. They are harbored in the outer leaflet of the membrane where their ceramide moieties partition them laterally into lipid rafts—spatially and temporally dynamic membrane microdomains that contain other sphingolipids, phospholipids, cholesterol, and specific proteins [28]. Several hundreds of ganglioside structures have been characterized based on differences in oligosaccharide chains or the fatty acids linked to the ceramide [29]. The total mass and specific ganglioside composition vary significantly between different cell types and tissues. The human brain contains up to 30-fold more gangliosides than other tissues [27], and they provide a significant part of neuronal surface glycans [30]. Four gangliosides—GM1, GD1a, GD1b, and GT1b—are the most abundant in the mammalian brain [31], with extraordinary regional pattern differences [32]. Gangliosides are synthesized stepwise by a vast number of glycosyltransferases in a complex biosynthetic pathway in the Golgi apparatus from where they are trafficked to the plasma membrane [33,34]. They play a crucial role in various physiological processes including brain development, aging, and neurodegeneration by modulating the cell signal transduction pathways, neuronal differentiation, and structures and functions of membrane proteins [26,35]. The functions of gangliosides in the central nervous system can be revealed by the physiological manifestations of genetic mutations in the ganglioside biosynthetic pathway, observed both from studies of rare human diseases resulting from mutations in the ganglioside biosynthesis pathway and transgenic mouse models with impaired synthesis of gangliosides [36,37]. 

Analogously to lipid-protein interactions, a general feature of gangliosides is their capability to associate laterally with other molecules within the membrane [38]. The hydrogen-bonding ability of their headgroups results in extensive *cis*- or *trans*- interactions with membrane proteins [31,39]. *Cis*-interactions with membrane proteins in the same membrane modulate their localization within membrane subdomains, e.g., lipid rafts [40,41,42]. Being lipid raft constituents, gangliosides are implicated in regulation of the activity of plasma membrane proteins, including protein tyrosine kinases. Many receptor tyrosine kinases, including epidermal growth factor receptor (EGFR), platelet-derived growth factor receptor (PDGFR), and insulin receptor (IR) are localized in lipid rafts, and their dimerization and/or recruitment of signaling partners are affected by gangliosides, thus influencing their respective signaling pathways [40,43,44,45]. Furthermore, there is evidence on regulatory effects of gangliosides on the function of the GluR2-AMPA receptor (AMPAR), crucial for learning and memory formation process [46]. Ganglioside GM1 was also shown to promote axonal growth by bringing together the TrkA and laminin-1/beta1 integrin complexes [47]. Ganglioside biology and physiological functions, particularly in mammalian brain, have been exceptionally well described by Schnaar et al. [31,36]. Undoubtedly, a large body of data supports the notion of gangliosides as essential molecular signatures of cell surfaces, where they function as ligands for various glycan-binding proteins, such as bacterial toxins, cell adhesion molecules and receptor kinases [25,40,43,44,45,46,47]. Despite comprehensive studies of ganglioside-protein interactions, the nature of these interactions is still not fully investigated and many details await elucidation.

### 2.2. Potential Role of Gangliosides in the Molecular Pathogenesis of Seizures

Epilepsy is a disease of the central nervous system characterized by pathological neuronal activity. Neuronal hyperactivity leads to abnormal, extensive, and uncontrolled symptomatology which can be presented as motor, sensory, cognitive, or autonomic disturbances. Pathological firing of neurons can have generalized onset, or focal onset with a possibility for bilateral tonic-clonic propagation or of the unknown onset [48]. There are many different causes of epileptic seizures [49], but the main feature of the seizure generation is either abnormal rhythmic and tonic excitation of neurons, or the aberrant interplay between inhibitory and excitatory neurons and altered membrane conductance [50]. Interestingly, several studies have associated mutations in ganglioside biosynthetic genes with epilepsy in humans. Mutations in the *ST3GAL5* gene, encoding for the GM3-synthase, result in an early-onset seizure disorder, also known as the salt and pepper syndrome, characterized by motor and cognitive disturbances [51]. GM3-synthase catalyzes the first step in the production of gangliosides by transferring a sialic acid residue to lactosylceramide to form GM3; thus, loss of its catalytic activity prevents the formation of GM3 and other complex gangliosides. Contrary to observed effects of altered GM3 synthase activity in humans, no neurological deficits but altered insulin responsiveness was found in a comparison study on the *St3gal5-null* mice [52], while mice expressing only the GM3 ganglioside exhibit lethal audiogenic seizures [53]. Furthermore, mutations in human GM3 synthase have been linked to early-onset symptomatic epilepsy syndrome associated with developmental stagnation, blindness, and mitochondrial dysfunction [54,55]. In a study on epileptic patients, quantitative changes in major ganglioside species have been reported, indicating increased expression and immunohistochemical localization of the GD3 ganglioside in the hippocampi of epileptic patients [56]. Ganglioside GD3 is normally present in negligible amounts in the adult human brain; therefore, finding an increase of GD3 in hippocampal tissue in epilepsy is quite compelling. Given that GD3 binds calcium ions with a high-affinity [57,58], accumulation of GD3 may increase the local concentration of extracellular calcium ions, resulting in hyperexcitability. West syndrome, an infantile epilepsy disorder associated with developmental arrest or regression, has also been linked to several mutations in genes involved in ganglioside biosynthesis. Decreased levels of gangliosides GM1 and GD1a were observed in affected patients [59]. Furthermore, mutations in the *ST3GAL3* gene, coding for ST3 beta-galactoside α-2,3-sialyltransferase 3, were observed in patients with West syndrome [60], and in two infants suffering from epileptic encephalopathy [61]. Since this enzyme catalyzes the sialylation of glycoproteins in addition to glycolipids in animal models [62], this may suggest that variations in the sialylation pattern on the surface of neuronal cells may cause subtle changes in tissue glycolipid composition that could result in the occurrence of epilepsy [60]. Deficits in another enzyme in the ganglioside biosynthesis pathway, the GM2/GD2 synthase encoded by the *B4GALNT1* gene, cause rare hereditary spastic paraplegia accompanied by intellectual and motor disabilities [63], concurrent with severe motor deficits, progressive dysmyelination, and axonal degeneration shown in *B4galnt1-null* mice [64,65,66]. Studies on mice lacking the GM2/GD2 synthase demonstrated an increased vulnerability of the mice to seizures and the ability of a semisynthetic analog of ganglioside GM1, LIGA 20, to attenuate seizures when administered intraperitoneally [67]. Administration of gangliosides GM1 and GT1b have been reported to stop induced convulsions in animal models [68,69], whilst higher levels of GQ1b ganglioside have been shown to cause seizures in amygdaloid kindling-mice [70]. In the study on amygdaloid kindling-mice, intracerebroventricular administration of total brain gangliosides worsened kindling seizures [71]. Other experimental evidence also points to a complex role of gangliosides in the pathogenesis of seizures. A study on epileptic rats [72] has shown a decrease of the total content of GM1, GD1a, GD1b and GT1b gangliosides, phospholipids, NKA activity and lipid peroxidation levels following seizures. These data indicate that seizure activity leads to changed lipid content and lipid peroxidation status, in addition to altered NKA activity. The authors suggest that all the stated parameters might contribute to the neurophysiopathology of seizures observed in patients suffering from epilepsy [72]. However, the question remains whether the changes in the quantity of particular gangliosides in the brain are a consequence or the actual cause of different types of seizures by modulating functions of membrane proteins involved in seizure pathogenesis.

## 3. Na^+^/K^+^-ATPase

The Na^+^/K^+^-ATPase (NKA), or the sodium-potassium pump, is a ubiquitously expressed plasma membrane protein belonging to the family of P-type ATPases. This group of enzymes use the energy from ATP hydrolysis to pump ions over the plasma membrane against their concentration gradient by autophosphorylation of key aspartate residue [73,74]. NKA pumps 3 Na^+^ out of the cell and 2K^+^ into the cell per single molecule of ATP hydrolyzed, thus maintaining a higher concentration of sodium ions outside and a higher level of potassium ions inside the cell. Maintenance of the ionic imbalance between the cell and its surroundings by NKA is of great importance for cell physiology, providing for the stabilization of the resting membrane potential by hyperpolarization, firing up the action potential, ion homeostasis, osmotic regulation of the cell volume, cell cycle and metabolism regulation, regulation of endosomal pH, and various intracellular signal transduction pathways [75,76,77,78]. Being involved in all the above-mentioned processes, it is not surprising that the amount of energy consumption by NKA in brain grey matter is about three-quarters of the total energy expenditure of the brain [78]. Structurally, NKA is a heterodimer of a catalytic α subunit and regulative β subunit needed for positioning the catalytic subunit within the membrane [79]. A member of the FXYD family is also known to interact with the αβ heterodimer [80] and different isoforms of particular subunits have been identified [73]. 

NKA is highly abundant in the brain and changes in its function and activity have been implicated in neurodegenerative disorders like Alzheimer’s disease (AD) [81], with its expression being decreased both in the brain tissue derived from AD patients and AD mouse models [82,83]. Interaction between the neuron-specific α3 subunit and patient-derived Aβ oligomers was found to have a neurotoxic effect causing neurodegeneration by presynaptic calcium overload, resulting in hyperactivation of neurons [84]. Mutations in the α subunits have also been found in individuals with other neurodegenerative disorders—the rapid-onset dystonia-parkinsonism (RDP), alternating hemiplegia of childhood (AHC), familial hemiplegic migraine (FHM), and hearing loss syndrome (CAPOS), all of which have overlapping symptoms including epileptic seizures, hyperactivity, dystonia, ataxia and cognitive deficits [85,86]. Contrary to the α subunits, no genetic neurodegenerative disorders are known to result from mutations in β and FXYD subunits [73]. 

### Na^+^/K^+^-ATPase Involvement in Epileptic Seizures

Ionic currents that arise from the ionic imbalance between the cell and the intracellular space maintained by NKA activity have been hypothesized to cause seizures by several mechanisms. A decrease in NKA activity increases neuronal excitability which could underlie the pathogenesis of seizures [87]. Reduction of NKA current was demonstrated as crucial for the stability of the physiological network, as the increase in NKA current mediated termination of seizures due to elevation of intracellular Na^+^ concentrations [88]. Partial inhibition of NKA activity by cardiac glycoside ouabain caused rapid, running, and leaping seizures when injected to rat hippocampi, whilst clonic-tonic seizures occurred when the drug was administered to the cerebellum and the brainstem [89]. Administration of dyhydroouabain and strophanthidin led to decreased extracellular K^+^ levels and increased Ca^2+^ conductance contributing to epileptic discharges in CA1 hippocampal regions of rats [90]. Strophanthidin was found to have different effects on NKA current between rat CA1 and CA3 regions, with higher depolarization observed in the CA3 region, suggesting the region is more adaptive to epileptiform events [91]. Depolarization of neuronal membranes as a consequence of decreased NKA activity was also observed in neurons derived from patients suffering from chorea-acanthocytosis, a rare neurodegenerative disorder with manifestations of epileptic seizures [92]. A decrease in NKA activity has been observed in the epileptic human cerebral cortex, proposing that an abnormal flux of cations may lead to disturbed electrolyte metabolism in the epileptic brain [93]. 

Furthermore, there are a significant number of studies linking mutations in NKA genes to different epileptic phenotypes. A mutation in ATP1A1 gene coding for the α1 catalytic subunit resulting in membrane depolarization was described in an infant suffering from lethal epilepsy [94]. Several mutations in ATP1A2 and ATP1A3 genes, encoding for α2 and α3 subunits, respectively, have been reported in a range of epileptic disorders, both in the brains of human subjects and mouse models [95]. FHM is a type of migraine with aura, with manifestations of epileptic seizures, cerebellar ataxia, and in more severe cases, coma. It was the first disorder to be linked to mutations in NKA subunits, as the loss of function of the α2 subunit was detected [96,97,98]. Functional studies have revealed some of the mutations in the gene to result in a change of NKA kinetic parameters, rather than the total loss of the enzymatic activity [99,100,101]. More recently, seven distinct mutations in ATP1A2 have been selected by causing different phenotypes in FHM patients, all of whom resulted in a similar level of NKA protein expression, but with decreased enzyme activity compared to the control groups, and with more severe phenotypes having the lowest NKA activity [102]. Loss of α2 function was detected in a cohort of seven patients with severe encephalic epilepsy carrying different mutations of the ATP1A2 gene [103]. A mutation has also been detected in two unrelated patients suffering from AHC, an early onset disorder outlined by epileptic seizures, ataxia, and learning disabilities [104]. Mutations in the ATP1A3 gene have been described in AHC as well, recognizing them as a key factor in AHC pathogenesis [105]. Several mutations in the gene resulting in a decrease of NKA activity were observed in children with AHC, but the mechanisms underlying the disorder remain unclear [106]. A study on an AHC mouse model carrying an α3 missense mutation caused similar phenotypes of AHC in human patients [107]. The importance of NKA in epilepsy was confirmed in the Myshkin mouse model, carrying a sole mutation in the ATP1A3 gene causing total loss of NKA activity, leading to epileptiform activity and seizures in these mice [108]. The differences in clinical manifestations of various epileptic pathologies are the result of different positions and natures of sole mutations as different amino acid substitutions occur. The diversity of structural changes in α subunits might also alter their ability to co-assemble with three β-isoforms and seven different FXYD domains, adding complexity to the elucidation of NKA involvement in epileptic seizures.

## 4. The Effects of Gangliosides on Na^+^/K^+^-ATPase

To elucidate the interplay between gangliosides and NKA, firstly, we must focus on their intrinsic qualities given by their structural features. Gangliosides achieve a series of intra- and inter-molecular interactions that shape lateral and spatial membrane organization [109,110,111]. The ceramide part of the molecule accomplishes gangliosides’ dense packing by accommodating cholesterols’ steroid moiety in between two neighboring molecules [109]. In addition, experimental and computational evidence of the effective negative charge abolishment of the first sialic acid carboxylate ion, termed NH-trick, contributes to heavy packing [109,112]. This was previously suggested by Rodriguez et al. [112] who described the existence of a hydrogen bonding interaction between the hydrogen atom of the non-ionized carboxyl group in *N*-acetylneuraminic acid (Neu5Ac) and the oxygen atom of the carbonyl group in *N*-acetylgalactosamine (GalNAc), which reduces the dissociation of the Neu5Ac carboxyl group. Furthermore, they also suggested a second hydrogen bonding interaction between the proton of the acetamide group in GalNAc and the carbonyl moiety of the carboxyl group of Neu5Ac.The spatial organization of the oligosaccharide part differs depending on the topology of the microdomain where chalice-shaped conformers accessorize the outer borders, and flat-surfaced conformers occupy the interior [109]. The membrane’s physical properties arising from these unique ganglioside characteristics include curvature, asymmetry, cooperativity, demixing, and metamorphism [90,91]. As gangliosides forge the local lipid environment in a range from nano- to micro-domain, they play a great role in setting up a functional environment for proteins associated with different membrane qualities. As a membrane protein, NKA demands specific lipids in its hydrophobic mismatch region. Although it encompasses a maximum of 33 annular lipids [113], the protein-lipid interface is populated with specific interaction partners revealed by mechanistic and structural studies involving NKA [113,114,115]. The interactive nature of NKA structural elements and membrane lipid constituents induces spatial stability and modulates ion-exchange frequency. Cholesterol can occupy two specific sites: (a) between the α and FXYD subunits, which favors stability, and (b) tightly squeezed between α and β subunits, disabling movement and pumping activity [114]. NKAs’ dependence on cholesterol is evident from systems with cholesterol-poor membranes, e.g., *Saccharomyces cerevisiae*, where NKA resides in an inactive state with no detectable enzymatic turnover [114]. Furthermore, neutral phospholipids of inner membrane leaflets were suggested to stimulate NKA activity [114], and sphingomyelin and saturated phospholipids inhibit it [114]. Conceptually, NKA’s enzymatic activity is a function of lateral lipid exchange within the annular region, and subtle changes of lipid species within NKA proximity can make enormous changes in the molecules’ activity status. These events of lateral lipid mobility are within gangliosides’ mixing-demixing ability [110], where gangliosides act as lipid sorting machinery providing the optimal *mise en scène* for a variety of proteins, including NKA. However, there have been only a minor number of studies on how gangliosides affect NKA enzymatic activity, performed on model or physiological systems. Our research group has established that NKA activity alternates during multiple freeze-thaw cycles due to reshuffling between lipid rafts and bulk membrane fractions accompanied by ganglioside GM1, and with GD1a, GD1b, and GT1b distribution mirroring the changes in NKA activity [116]. These results indicate that gangliosides arrange a distinct lipid environment crucial for proper NKA functioning. Hence, as opposed to investigating the effects on NKA by exogenously adding gangliosides, within real membranes the change of ganglioside composition is achieved between distinct membrane domains. Considering that different gangliosides populate different lipid rafts in various cell types [117], this could be an important means of fine-tuning NKA activity. Furthermore, described pools of non-pumping NKA in epithelial cells are a consequence of NKA positioning within membrane domains with different cholesterol concentrations, caveolae and lipid rafts [118]. Extensive NKA mobility in neuronal membranes [119] could be ganglioside driven, thus utilizing proteins’ different modes of operation and maintaining neuronal membrane homeostasis. Ganglioside-NKA interactions were also investigated by electron spin resonance (ESR) spectroscopy using spin-labeled gangliosides GM1, GM2, GM3, and GD1b and NKA purified from shark rectal glands, in conjunction with other charged membrane lipids. The results indicated gangliosides GM1, GM2, and GM3 exhibit virtually no selectivity in interactions with NKA relative to spin-labeled phosphatidylcholine, whilst GD1b selectivity was considerably lower relative to other phospholipids, possibly due to one more sialic acid residues in its structure [120]. The authors propose that this low selectivity is due to the bulkiness of ganglioside head groups which prevents closer associations with the protein. 

Up to today, most of the studies on how gangliosides affect NKA activity have investigated the effects of the addition of exogenous gangliosides on NKA activity in various types of tissue preparations originating from different species, often resulting in contradictory results (Table 1).

Total brain gangliosides isolated from porcine brains reduced NKA activity up to 40% in chicken brain synaptosomal preparations when ganglioside-protein ratios were 1:1, with the kinetics described as a non-competitive inhibition, and the interactions being dependent on temperature, ATP concentration, and preincubation time [121]. Interestingly, the increase in ganglioside concentrations was found not to affect NKA activity in synaptosomal fractions derived from cats suffering from GM1 and GM2 gangliosidosis [122]. In another study on rat brain microsomal preparations, administration of total human brain gangliosides had opposite effects regarding their concentration. The lower concentrations were shown to activate and higher to inhibit NKA activity, with the inhibition being reversible and competitive relative to K^+^ concentrations [123]. The authors suggest that the inhibition of NKA activity results from conformational changes in the membrane caused by the integration of exogenous gangliosides. In another study, microsomal fractions from adult male rats were treated with total brain gangliosides and various sphingolipid species derived from a patient with Tay–Sachs disease resulting in slight activation of NKA activity [124]. Furthermore, the addition of nanomolar concentrations of exogenous GM1, GD1a, GD1b, GT1b gangliosides to mitochondrial fractions derived from rat brains caused a 26–43% rise in NKA activity under identical conditions, with authors suggesting the phenomenon to be directly linked to alternations in the enzyme’s lipid environment [125]. All the above findings reflected in NKA activity alterations affirm that gangliosides affect the membrane in a different manner depending on membrane specialization and complexity. Ganglioside GM1 itself was found to cause minor inhibition of NKA activity when added to homogenates prepared from rat superior cervical ganglion (SCG) [126,127], but an increase in the activity in nodose ganglia (NG) from adult rats [127,128]. Administration of ganglioside GM1 has been shown to have potential therapeutic properties since it was able to decrease mortality due to ischemia up to 52% in Mongolian gerbils. However, no significant differences were observed in NKA activity between occluded and non-occluded brain hemispheres [129]. The same group has again observed neuroprotective action of GM1 in comparison to the saline treatment of induced ischemia in the gerbil cerebral cortex [130]. In another study, striatal homogenates derived from rat brains injected with convulsion-causing glutaric acid (GA) and pentylenetetrazole (PTZ) were incubated with GM1. A decrease in NKA activity resulting from GA administration was shown to cause seizures in vivo, and an ex vivo decrease in NKA activity to be prevented by GM1 administration, suggesting its possible therapeutic application in the treatment of seizures [68]. More recently, ganglioside GM1 was also found to have neuroprotective properties in hippocampal homogenates from AD models, as it was able to prevent the decrease in NKA activity caused by the Aβ1-42 oligomer administration [133]. GM1 administration was also shown to reduce the effects of hypoxia-reoxygenation in rat heart, as it was able to stabilize NKA activity which was decreased due to hypoxic conditions [131]. In addition, a complete correction of a decrease in NKA activity in nerve homogenates from diabetic rats treated with mixed bovine gangliosides was observed over a five-week period [132].

## 5. Conclusions

**Figure 1:**
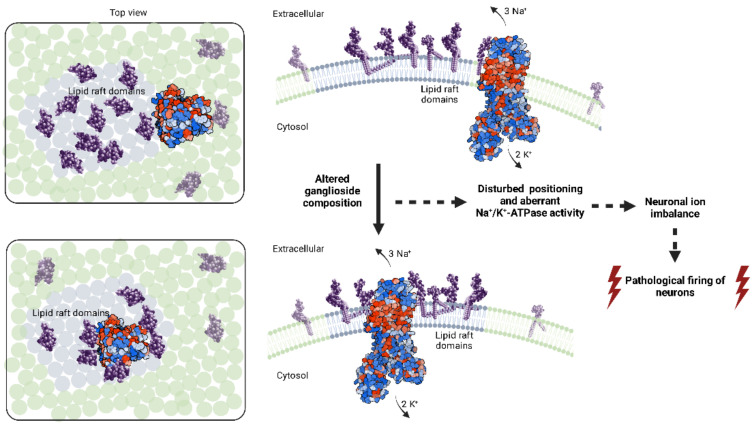
Schematic representation of sequence of events leading from altered ganglioside composition of the neuronal membrane to pathological firing of neurons and epileptic seizures. Na^+^/K^+^-ATPase (NKA) positioning is heavily influenced by the surrounding gangliosides. NKA is represented by Biorender.com (accessed on 26 April 2022), relying on PDB accession code 3B8E, according to [134], utilizing the Van Der Waals structure style and Hydrophobicity color style. Red color represents amino acids with more hydrophobic side chains, and blue represents amino acids with more hydrophilic side chains. Gangliosides (in space-filling model) are shown in purple.

The purpose of this review was to map out the many possible points of convergence between gangliosides and NKA, which in tandem lead to the development of epileptic seizures if their respective functions are disturbed. The presented evidence may serve as a direction for new experimental approaches to investigate the observed phenomena in detail. We explore a select facet of neuronal membrane dynamic environment which may have crucial impact on ion homeostasis and pathological ion imbalance. We propose that specific sets of gangliosides and NKA possibly form highly functional assemblies within membranes. We summarize the overwhelming experimental evidence on the intricate relationship between NKA and gangliosides, which includes: (i) Exogenously added gangliosides have a measurable effect on NKA activity. The demonstrated change in activity of NKA is found to be concentration-dependent and specific ganglioside species exert different responses in NKA activity [121,122,123,124,125,126,127,128,129,130]; (ii) Ganglioside-NKA interactions studied by electron spin resonance spectroscopy show differential selectivity in interactions between gangliosides and NKA [120]; (iii) Impaired NKA activity is restored following ganglioside treatment in models of different pathological conditions, including seizures caused by glutaric acid administration, Alzheimer’s disease model, hypoxic conditions and diabetes [68,131,132,133]. Certain neuroprotective effects of specific gangliosides are suggested to be mediated through modulation of NKA activity [133]. We therefore argue that structural changes of neuronal membrane caused by altered ganglioside composition most likely contribute to aberrant NKA activity, with the resulting ion imbalance priming neurons for pathological firing (Figure 1).

The existence of specific functional interaction(s) between gangliosides and NKA, is of immense significance for properties of neuronal membranes, and is strongly corroborated by different epileptic phenotypes in humans and murine models caused by either impaired activity of NKA or changed membrane ganglioside composition. Clarifying the exact effect of gangliosides on NKA activity may greatly contribute to understanding the complexity of well-regulated molecular events involved in maintenance of neuronal membrane ion homeostasis. Since the administration of gangliosides to patients suffering from various neuropathological conditions is being intensely investigated [135,136], the research avenues which would take into account the suggested NKA-gangliosides partnership may reveal new molecular targets in diagnosis and treatment of epilepsy. 

## Figures and Tables

**Table 1:** Summarized data describing the effect of exogenously added gangliosides on NKA activity.

Animal Model	Sample Type	Gangliosides Studied	NKA Activity	Publication
Chicken	Brain synaptosomes	Total porcine brain gangliosides	↓	[121]
Cat	Brain synaptosomes	GM1	No change	[122]
Rat	Brain microsomes	Total human brain gangliosides	↑↓ *	[123]
Rat	Brain microsomes	Total human brain gangliosides	↑	[124]
Rat	Brain mitochondrial fractions	GM1, GD1a, GD1b, GT1b	↑	[125]
Rat	SCG homogenates	GM1	↓	[126,127]
Rat	NG homogenates	GM1	↑	[127,128]
Mongolian gerbil	Brain homogenates	GM1	No change	[129,130]
Rat	Striatal homogenates	GM1	↑	[68]
Rat	Heart	GM1	↑	[131]
Rat	Nerve homogenates	Mixed bovine gangliosides	↑	[132]

* lower concentrations were shown to activate and higher to inhibit NKA activity. SCG = superior cervical ganglion, NG = nodose ganglia.

## Data Availability

Not applicable.
